# Bioprocessing Considerations towards the Manufacturing of Therapeutic Skeletal and Smooth Muscle Cells

**DOI:** 10.3390/bioengineering10091067

**Published:** 2023-09-09

**Authors:** Teresa Franchi-Mendes, Marília Silva, Ana Luísa Cartaxo, Ana Fernandes-Platzgummer, Joaquim M. S. Cabral, Cláudia L. da Silva

**Affiliations:** 1 Department of Bioengineering, iBB—Institute for Bioengineering and Biosciences, Instituto Superior Técnico, Universidade de Lisboa, Av. Rovisco Pais, 1049-001 Lisboa, Portugal; maria.franchi.mendes@tecnico.ulisboa.pt (T.F.-M.); mariliamsilva@tecnico.ulisboa.pt (M.S.); luisa.cartaxo@tecnico.ulisboa.pt (A.L.C.); ana.fernandes@tecnico.ulisboa.pt (A.F.-P.); joaquim.cabral@tecnico.ulisboa.pt (J.M.S.C.); 2 Associate Laboratory, i4HB—Institute for Health and Bioeconomy, Instituto Superior Técnico, Universidade de Lisboa, Av. Rovisco Pais, 1049-001 Lisboa, Portugal

**Keywords:** skeletal muscle cells, smooth muscle cells, tissue engineering, cell manufacturing

## Abstract

Tissue engineering approaches within the muscle context represent a promising emerging field to address the current therapeutic challenges related with multiple pathological conditions affecting the muscle compartments, either skeletal muscle or smooth muscle, responsible for involuntary and voluntary contraction, respectively. In this review, several features and parameters involved in the bioprocessing of muscle cells are addressed. The cell isolation process is depicted, depending on the type of tissue (smooth or skeletal muscle), followed by the description of the challenges involving the use of adult donor tissue and the strategies to overcome the hurdles of reaching relevant cell numbers towards a clinical application. Specifically, the use of stem/progenitor cells is highlighted as a source for smooth and skeletal muscle cells towards the development of a cellular product able to maintain the target cell’s identity and functionality. Moreover, taking into account the need for a robust and cost-effective bioprocess for cell manufacturing, the combination of muscle cells with biomaterials and the need for scale-up envisioning clinical applications are also approached.

## 1. Introduction

Even though a considerable progress has been made in the cell therapy field for muscular disorders, there is still significant clinical demand for tissue-engineered muscle for transplantation or replacement therapy [1]. Indeed, tissue engineering approaches hold a promising future in treating diseases that affect skeletal muscle (SkM) and smooth muscle (SM), including cases of muscular dystrophies and volumetric muscle loss (VML) after cancer or trauma [2]. Moreover, muscle tissues that rely on contractile activity, such as the sphincters, bladder, intestine, diaphragm, face, hand, tongue, pharynx, larynx and esophagus, could potentially also be restored, or replaced using tissue engineering approaches [3]. Epidemiology of disorders related to SkM and SM are difficult to truly ascertain, as they can be multisystemic. For instance, reports on Duchenne muscular dystrophy (DMD), a SkM genetic disease, point to 3–7 cases per 100.000 population [4]. On the other hand, reports on SM-related disorders, such as stress urinary incontinence, suggest a prevalence of 30–40% in women [5,6], but these numbers may be underestimated due to the social stigma associated with them.

Tissue engineering approaches can include the use of stem/progenitor cells, combined with appropriate biomaterials, to generate the suitable microenvironment to functionally repair, replace and regenerate the damaged or lost organ [7,8]. From this perspective, to provide clinically relevant engineered muscle tissues, there is a demand for an in-depth optimization of the manufacturing of their functional building blocks, either skeletal muscle cells (SkMCs) and/or smooth muscle cells (SMCs). Importantly, a critical point in what concerns the engineering of these tissues is the need to generate functional cells with a contractile phenotype [9]. 

SM is derived from both mesoderm and neural crest cells, and it can have a local common progenitor origin in adult tissue (for example, vascular progenitors) [10]. SM tissue is located throughout the body and is crucial, from a functional standpoint, in a variety of tissues [11]. For instance, in the gastrointestinal tract, SM is essential for motility; therefore, any damage to the SM of the gastrointestinal system may have severe effects on digestion and nutrient absorption. In the urinary system, at the level of the kidneys, vascular SM dysfunction is associated with chronic kidney disease and can lead to end-stage renal disease [12]. In the cardiovascular system, SM is present in vessels to maintain blood pressure and flow, whereas in the respiratory tract, it is responsible for opening and closing airways. Overall, SM serves a purpose in almost every other organ system [11]. At a cellular level, SM is described as a nonstriated muscle, with neural innervations from the autonomic nervous system, and it differs from SkM in many ways, possibly the most functionally significant being its ability to be contracted and controlled involuntarily [11]. SMCs are usually characterized by identification of multiple markers namely smooth muscle actin (α-SMA), smoothelin, calponin, smooth muscle 22 (SM22α), and smooth muscle myosin heavy chain (MYH11) [10,13]. These proteins can also be transiently detected in other cell types, such as α-SMA in activated fibroblasts or myofibroblasts [10]. Therefore, in vitro manufacturing of SMCs needs to take into account the multitude of markers for SMC identity, as well as the ability for contractility.

When it comes to SkM, it represents about 40% of the human body mass and it is composed of bundles of voluntary contractile multinucleated muscle fibers, resulting from the fusion of myoblasts [2]. SkM is one of the adult tissues that still holds a remarkable ability to regenerate itself in response to injury (as well as exercise), despite the post-mitotic nature of its myofibers, due to the presence of a primitive cell population, defined as satellite cells [14]. This population is recognized as SkM-resident stem cells, located between the plasma membrane of myofibers and the basal lamina, providing a homeostatic microenvironment for tissue regeneration [14]. However, in cases of severe injury consisting of VML, the damage cannot be repaired naturally, affecting the patients’ quality of life by seriously limiting musculoskeletal functionality [15]. Causes of SkM damage are traumatic injuries; tumor resections and degenerative genetic diseases, namely DMD; amyotrophic lateral sclerosis (ALS); and pediatric Charcot–Marie–Tooth disease [15]. Mitotically quiescent satellite cells are activated once a disruption in the myofiber occurs as a response to muscle lesion [16,17]. Once activated, satellite cells undergo asymmetric division [18,19]. This maintains a satellite cell pool and generates myoblasts, which in turn proliferate and differentiate, giving rise to multinucleated myotubes [19,20]. During the myogenic process, there is a tight temporal regulation by specific transcription factors [21]. Particularly, satellite cells express paired box transcription factor 7 (Pax7), while myogenic differentiation factor 1 (MyoD) is expressed during myoblast proliferation, and myogenin is highly expressed once the differentiation into myocytes occurs [17,21]. Muscle-specific proteins, such as Desmin, a type III intermediate filament; sarcomeric α-actinin; and contractile proteins myosin heavy-chain 1 and 2, are typical identifiers of the terminal differentiation stage for SkMCs [17].

Although the myogenic process has defined hallmarks, it involves complex temporal dynamics and a mix of cell populations [22]. Firstly, satellite cells are considered a heterogeneous population [23] and consequences on their functionality are not yet fully understood. For instance, Pax7 transcription factor is the classic marker for satellite cells, but a population of Pax7-negative human muscle-derived cells able to regenerate muscle after transplantation in mouse models of muscle damage was identified [24]. On the other hand, the muscle milieu comprises not only myogenic cells, but also nonmyogenic players that have an essential role in homeostasis and during muscle lesion and repair [25]. Upon muscle injury, for example, immune cell infiltration (macrophages and neutrophils) occurs, creating firstly a proinflammatory environment that sustains satellite cell proliferation, followed by a balance of anti-inflammatory factors, which in turn favor a microenvironment towards differentiation [22,26,27]. Moreover, fibroadipogenic progenitors have been identified within the muscle milieu and cause crosstalk during muscle injury [28]. In this situation, they support myoblast differentiation and can also differentiate into myofibroblasts, which secrete the extracellular matrix (ECM) that surrounds the new myofibers [28]. Fibroadipogenic progenitors can also interact with immune cells, with a reciprocal regulation [28]. Furthermore, a population of denominated muscle-derived stem cells (MDSCs) has been reported as co-expressing myogenic and endothelial cells (ECs) markers, exhibiting myogenic differentiation potential in vitro and in vivo [29,30]. However, it is not clear if they represent an intermediate state during myogenesis or if they refer to the same population, only varying due to differences in isolation method [17].

As highlighted above, tissue engineering approaches for SM and SkM regeneration are of utmost clinical significance due to the broad functional relevance of these tissues throughout the body. As such, it is imperative to explore bioengineering strategies towards the development of advanced regenerative therapies to restore muscle structure and function.

## 2. Isolation and Ex Vivo Expansion of Smooth and Skeletal Muscle Cells

### 2.1. Smooth Muscle Cells (SMCs)

**Figure 1:**
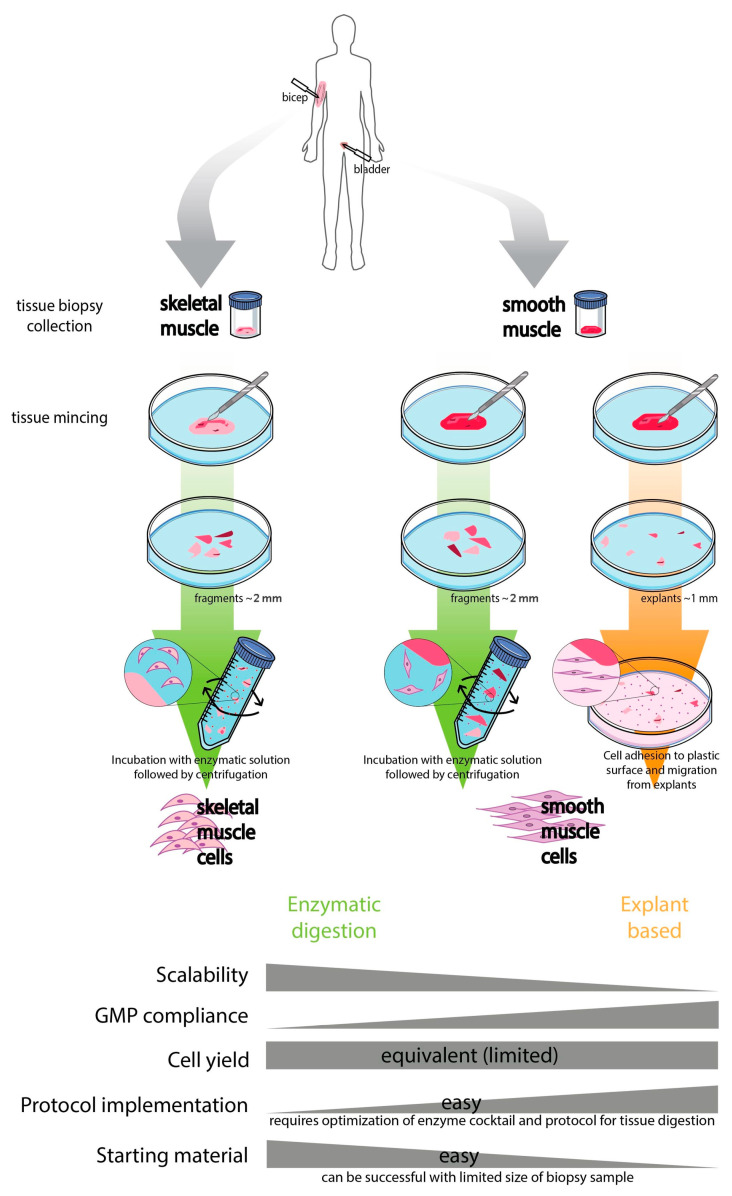
Schematic representation and comparison of isolation methods for primary SkMCs and SMCs: enzymatic digestion and explant-based approaches. Enzymatic-based methods for SkM and SM processing involve tissue sample collection, followed by tissue mincing, digestion with proteases (e.g., collagenase and/or dispase enzymes), filtering through a cell strainer, resulting in a cell suspension that is usually plated on coated surfaces for SkMCs (such as Matrigel) and on plastic surfaces for SMCs. Another cell isolation method is the explant-based protocol, which is mainly applied for SMCs, while for SkMCs, it is highly limited and has been mainly purposed towards tissue and disease modelling. The explant technique involves fragmentation of the tissue sample into approximately 1–2 mm diameter explants, followed by cell adhesion, migration and proliferation from the explants onto the plastic surface. Each isolation approach is compared in different categories: scale-up is easier when using enzymatic approaches, while explant-based can facilitate good manufacturing practice (GMP) compliance and simpler protocol optimization. As enzymatic approaches comprise the selection of enzyme(s) composition, concentration and digestion duration, there is a need for a balance between milder digestion protocols and insufficient cell retrieval in contrast to more harsh protocols that can result in higher cell numbers, but with limited viability. Although the resulting cell yield from both explant and enzyme-based methods is described as limited, enzymatic methods can be advantageous when having a reduced amount of starting sample. Adapted from [17,56,57]. GMP: good manufacturing practice; SkMCs: skeletal muscle cells; SMCs: smooth muscle cells.

SMCs are an essential cell type found in several organs, including the respiratory tract, gastrointestinal tract, urinary bladder, uterus, male and female reproductive tracts and the vascular system [31]. Methodologies for SMC isolation described in the literature are based on explant and enzymatic digestion techniques (as depicted in Figure 1), mainly from human, porcine and rodent bladder tissues [32]. In the explant method, cells are allowed to adhere and migrate from the explant onto the culture surface followed by proliferation. The second method involves enzymatic digestion of the tissue sample followed by plating the dispersed cells onto a surface for adherent cell culture [32]. Contamination of primary cultures of SMCs with fibroblasts constitutes a major drawback due to their potential to outgrow the target cells. Both SMC isolation protocols were compared in terms of robustness and efficiency envisaging tissue engineering applications. In a study by Pokrywczynska et al., the most homogenous culture (98% purity) was obtained when porcine SMCs from bladder tissue were isolated with collagenase and dispase digestion [32]. Moreover, the enzymatic methods utilizing collagenase and dispase, and collagenase alone, enabled the isolation of a significantly higher number of viable cells compared to explant techniques [32]. Studies have been published outlining the procedures for isolating vascular SMCs, employing both explant and enzymatic digestion methods [33]. Successful culture of vascular SMCs using enzymatic digestion relies on critical factors such as the specific enzymatic composition and the duration of digestion. These factors may vary depending on the source of vascular tissue and the specific vascular bed under investigation, as the type and amount of connective tissue can differ across samples. The enzymatic cocktail described by Ray et al. is advantageous, as it allows for the rapid and reproducible isolation of vascular SMCs from murine aorta where large amounts of starting tissue might not be readily available [34]. Other studies also described the use of enzymatic cocktails based on collagenase only or collagenase and elastase for isolating vascular SMCs from rat model tissues [35,36]. Moreover, magnetic forces were employed to facilitate tissue digestion and, importantly, SMC phenotype was validated through the identification of multiple markers (α-SMA, smoothelin, calponin, SM22α and MYH11) and functional assays [35]. In contrast, using explants, McMurray et al. reported a standardized method for culturing aortic explants to study factors affecting phenotypic modulation of cells in culture [36]. These authors suggested that explant cultures provided a system for studying the growth of vascular SMCs without fully digesting the tissue, thus circumventing the variability issues associated with enzymatic digestion [37]. 

Overall, the challenges associated with SMC isolation methods rely on the type of protocol (enzymatic- or explant-based), which can impact the cell number obtained. Additionally, this also depends on the amount and quality of the tissue source (vascular, nonvascular). Therefore, alternative approaches comprising the use of induced pluripotent stem cells (iPSCs), as well as mesenchymal stromal cells (MSCs), are also being explored to achieve clinically significant cell numbers, which is covered in Section 3.1. Importantly, regardless of the cell isolation procedure and tissue source, SMC identity and functionality need to be carefully addressed throughout the culture process.

### 2.2. Skeletal Muscle Cells (SkMCs)

Cell isolation from different anatomic SkM groups has been attempted, including deltoid, triceps, quadriceps, sternocleidomastoid or vastus lateralis. Satellite cells represent about 3–5% of the nucleated cells in adult human muscle, and this number tends to decrease with aging [38]. Muscle progenitors (i.e., myoblasts) have been isolated through tissue mincing and enzymatic digestion using collagenase and dispase [39,40], as schematically depicted in Figure 1. This method displays limited efficiency not only due to the low content of primitive cells in adult muscles, but also due to the need to pass the resultant suspension through cell strainers to remove nondigested tissue [40]. Furthermore, as it occurs with SMCs isolation, fibroblast contamination can occur along culture time. Besides enzymatic digestion, explant or single myofiber isolation methods have also been attempted for several years, although these display a high inefficiency for the isolation of SkMCs (and subsequent ex vivo expansion) and have been mainly purposed towards pathophysiology models, namely to study muscle electrophysiology [41,42]. Nonetheless, isolated muscle progenitors can usually be plated onto collagen- [43,44] or Matrigel- (a basement membrane extract from mouse sarcoma) coated surfaces [45]. 

In terms of absolute cell numbers retrieved after isolation based on enzymatic protocols, approximately 1–2 × 10^5^ myogenic progenitors could be collected from a single murine muscle [46], while combined protocols involving tissue digestion and explant outgrowth onto Matrigel have resulted in 1–2 × 10^7^ myoblasts [40]. Moreover, in terms of culture medium conditions, it has been observed that isolated satellite cells of murine origin seem to retain their quiescent state when cultured under low serum conditions (2%) and proliferate when using high serum-content medium (20%) [47], which can constitute an artificial stimulus of muscle injury. These primitive cells can proliferate in vitro and preserve levels of myogenic differentiation potential, with identification of myotube formation capacity [45,47]. 

Cell enrichment using fluorescence/magnetic-activated cell sorting (FACS/MACS) has been described, for example, in murine models, through which muscle stem cells have been purified using VCAM^+^CD31^−^CD45^−^Sca1^−^ sorting [48]. Sacco et al. reported the isolation of murine satellite cells from tibialis anterior muscle using enzymatic digestion and FACS enrichment based on a combination of markers: lack of expression of CD45, CD11b, CD31, Sca1 and positivity for CD34/integrin-α7 [23]. This enriched cell fraction has shown potential for transplantation, as cells proliferated and integrated into myofibers in recipient mouse muscle upon injury, as verified by tracking of Pax7^+^ mononucleated cells using luciferase and bioluminescence techniques [23]. In another study, a myosphere culture of human SkM-derived stem cells was established, with cells isolated through enzymatic digestion (from omohyoid muscle) without the need for Matrigel [49]. Despite the advanced age of the muscle biopsy donors (60 years old), the cells exhibited proliferative capacity over several passages and were able to differentiate into multinucleated myofibers [49]. In the comprehensive study conducted by Garcia et al., successful isolation of satellite cells from different human muscle biopsies (gastrocnemius, latissimus, vastus lateralis, rectus abdominis, among others) was reported [50]. Although there was a variable degree on the cell yield obtained per muscle, the authors typically achieved 10^4^ highly purified satellite cells per 1 g of adult SkM. It is worth noting that the number of cells per gram presented a decreasing trend towards donors aged over 80 years old, which is in line with other reports of satellite cell deregulation during aging [51]. The complex isolation protocol established by Garcia et al. involved enzymatic digestion, MACS and FACS. Briefly, a negative selection using magnetic beads was performed to remove CD31 and CD45 positive cells, followed by flow cytometry purification of the CD31^−^/CD34^−^/CD45^−^/CXCR4^+^/CD29^+^/CD56^+^ population. Interestingly, the therapeutic potential of these isolated cells was assessed by injection into immunocompromised mice with muscle injury and mouse models of DMD, being observed that human-derived Pax7 cells generated myofibers in the damaged muscles. Importantly, the isolated human cells retained their skeletal phenotype even after cryopreservation and thawing [50]. By using the explants technique, followed by MACS enrichment for CD56^+^ cells and subsequent culture in Matrigel and collagen constructs, it was observed that the CD56^+^-enriched fraction generated more myotubes compared to the unsorted counterparts [52]. The CD56-negative fraction mainly consisted of interstitial fibroblasts, along with smaller percentages of MSCs, immune cells, fibroadipogenic progenitors and ECs. The authors also tested an in vitro chemical muscle injury, which resulted in decreased hydrogel deformation as a measure of myofiber functionality [52]. This observation was then followed by proliferation of Pax7+ and MyoD^+^ cells, which identify satellite cells and committed progenitors, respectively [53].

Taken together, expansion of tissue-derived SkM stem/progenitor cells can be extremely time-consuming, and cost-effective approaches need to be established. Challenges in this field include (i) limited cell source (low percentage of satellite cells in adult SkM); (ii) lack of robust cell isolation protocols that allow for the collection of sufficient cell numbers; (iii) limited expertise in implementing scalable expansion platforms; (iv) in vitro expanded satellite cells potentially exhibiting impaired ability for muscle engraftment in vivo [54]. More specifically, satellite cells and myoblasts exhibit restricted proliferation ex vivo, tend towards spontaneous differentiation or enter a senescent phenotype, limiting their expansion potential even more [16,24,55].

## 3. Strategies for Advancing SMC and SkMC Manufacturing

The production of engineered tissues and organs requires the use of a large number of cells. However, a major challenge in what concerns the therapeutic application of SMCs and SkMCs relies on their low amount in the tissue sources, resulting in insufficient quantities of isolated cells for clinical use. Even though autologous treatments may have a lower risk of rejection, it may not be feasible for all patients due to limitations related to cell availability and impaired cell fitness. In this context, allogeneic cell manufacturing, in large-scale setups, holds great potential in the muscle regeneration field. As such, the identification of effective expansion techniques is the first crucial step to obtain the required number of functional cells in a time- and cost-effective manner. Section 3.1 and Section 3.2 describe strategies for improving the in vitro/ex vivo expansion of SMCs and SkMCs, testing multiple cell sources and different culture settings (e.g., culture medium composition, biomaterial). These are also summarized in Table 1 and Table 2 for SMCs and SkMCs, respectively.

### 3.1. SMCs

As aforementioned, one of the major limitations of cell-based regenerative therapy targeting SM is the lack of donor tissue suitable for cell harvesting. For this reason, the use of stem cells, namely iPSCs, as a source of therapeutic SMCs has generated increased interest in the field. In particular, human-induced pluripotent stem cell (hiPSC)-derived vascular smooth muscle cells (VSMCs) are of great value for disease modeling, drug screening, cell therapies, and tissue engineering, offering an innovative approach to replacing or bypassing diseased blood vessels [58]. Considering the great challenge of retrieving enough primary VSMCs from fetal or adult human tissues, hiPSCs are considered as a valuable resource due to their easy accessibility, expandability and ability to give rise to almost any desired cell type [59]. Also, patient-specific derived iPSCs retain the patient’s genetic information, allowing these cells to trigger little to no immune response after transplantation [59]. Still, protocols to efficiently produce high quantity of hiPSC-derived VSMCs need further optimization. From this perspective, a scalable method for manufacturing this cell type was developed using alginate hydrogel microtubes, which resulted in high viability, purity (>80%) and yield (~5.0 × 10^8^ cells/mL) [60]. The alginate hydrogel offers protection from hydrodynamic stress and limits cell mass to less than 400 µm, ensuring efficient nutrient diffusion while also reducing cell agglomeration [60]. Moreover, bioreactor-expanded VSMCs contributed to blood vessel formation in vivo, while also retaining similar expression levels of VSMCs markers compared with 2D cultured VSMCs [60]. Also aiming to improve hiPSC-derived VSMC production, Fang et al. proposed a hypoxic (5% O_2_ ) treatment during differentiation, effectively inducing proliferative hiPSC-derived VSMCs, via embryoid body-based differentiation [61]. The hypoxic conditions enhanced the formation, adhesion and amplification rates of embryoid bodies, and upon directed differentiation, hiPSC-VSMCs exhibited increased cell viability compared to culture under atmospheric air [61]. Envisaging the application of cell or tissue-based products in clinical practice, it is imperative to adapt protocols towards xeno(geneic)-free conditions, since animal-derived reagents may carry zoonoses and trigger immune responses of cell or tissue derivatives, which could lead to graft failure, besides being associated with batch-to-batch variability and lack of standardization [62]. From this perspective, a combination of human serum and human platelet lysate demonstrated effectiveness in replacing fetal bovine serum (FBS) to generate VSMCs from hiPSCs [58]. Functional xeno-free hiPSC-derived VSMCs were successfully obtained, suitable for scaffold-assisted vascular tissue engineering, which exhibited comparable mechanical strength to those developed from xenogeneic hiPSC-derived VSMCs [58]. This finding is consistent with the application of both human serum and human platelet lysate as substitutes for FBS in expansion strategies for cell therapies and tissue engineering [62,63,64]. To further improve cell purification from iPSCs, Li et al. used sorting (MACS or FACS) for CD34-positive cells to enrich for common vascular progenitors, followed by differentiation towards SMCs using platelet-derived growth factor (PDGF-BB) as medium supplement [65]. The obtained SMC population was injected into mouse models of urinary incontinence and the authors observed tissue remodeling with higher detection of elastin in the bladder [65].

Another cell type investigated as a source to obtain differentiated SMCs are MSCs, particularly those derived from adipose tissue (also referred to as adipose-derived stem cells (ASCs)). Chemical, physical and biological cues can be used to drive stem cell fate in vitro [17]. The suitability of three-dimensional scaffolds for culture and differentiation of MSCs can be influenced by the physical properties of the scaffold. This includes surface topography, microstructure and mechanical specifications, which affect cell adhesion, proliferation and differentiation [66]. Moreover, the use of microcarriers combined with stirred bioreactors is a widely applied technique for expanding anchorage-dependent cells [67,68]. The microcarriers provide a large surface area for cell adhesion and growth in a homogeneous and controlled environment. Envisioning a protocol to expand and differentiate ASCs into SM-like cells, and exploiting a format that requires minimal manipulation before clinical delivery, Parmar et al. prepared a microcarrier formulation composed of a biocompatible and degradable material, poly (D,L lactic-co-glycolic acid) (PLGA), using a thermally induced phase separation (TIPS) technique [69]. This resulted in a highly porous structure that facilitated controlled degradation compared with solid microcarriers. The authors observed that ASCs readily attached to the surface of TIPS microcarriers, differentiating into an SM-like phenotype. Moreover, expansion of SMCs on the surface of the microcarriers did not alter the integrity of the polymer microspheres, making them suitable as a potential cell delivery vehicle [69]. Using a similar experimental design, Ahmadi et al. also observed that SMCs can attach to PLGA microcarriers in suspension culture and exhibited enhanced cell growth combined with increased cell release capacity at the sites of delivery [70]. By using ASCs combined with collagen in a microsphere format, Walters et al. observed that SMC morphology and identity markers were highly detected under the presence of growth factors, namely PDGF-AB and transforming growth factor (TGF-β1), and under mechanical stretch [71]. In another study, ASCs were also used as a source for SMCs, without any scaffolds, under differentiation culture conditions using a low percentage of serum (1% FBS) [72]. SMC markers were identified at the end of 3 and 6 weeks in culture, by detection of α-SMA, MYH11 and smoothelin at protein and mRNA levels. Still, further elucidation is needed regarding the cell numbers obtained, for example, per gram of initial sample of adipose tissue, as well as the functionality of the generated SMCs [72].

When targeting the development of more robust methods for muscle tissue manufacturing, it is not only crucial to design effective platforms for cell expansion, but one should also consider that engineered SM tissues should be constructed with well-differentiated and aligned SMCs for proper functioning, mimicking native tissue. Keeping this in mind, organized cell/scaffold hybrids were employed as functional SM constructs using a bioreactor system [73]. Briefly, prior to bioreactor expansion, cells were seeded into porous sheet-type scaffolds, fabricated with polyurethane, and then subjected to cyclic mechanical strain with a self-designed stretching chamber. It was possible to conclude that cells had proliferated effectively after a 7-day culture period in the bioreactor, retaining their native premodulated contractile characteristics [73]. Moreover, mechanical stimulation is particularly critical for engineered vascular tissue, where in vivo shear forces at the blood interface help maintain the function of the endothelium [74]. In fact, various studies have shown that mechanical stresses are essential for growing tissue-engineered vascular constructs [75]. In this context, Mun et al. associated a pulse bioreactor system with poly (lactide-co-caprolactone) electrospun scaffolds to generate a 3D tubular-shaped graft, which exhibited mechanical properties similar to native blood vessels [76]. The SMCs expanded in this platform retained their basic characteristics, namely presence of α-SMA, and presented higher proliferation rates under pulsatile flow than when compared to static culture. Moreover, the physical stimuli comprising medium flow and pressure through the lumen of the construct enabled mimicking aspects of the native physiological environment [76]. In an attempt to mimic a vascular tunica media graft, electrospun gelatin fiber scaffolds were developed to grow human umbilical vein SMCs, making it possible to obtain SM-like cells with high viability upon expansion in a bioreactor system [77]. In fact, the dynamic setup almost doubled the rate of cell proliferation through the scaffold compared to static conditions, forming a full tissue-like structure throughout a scaffold of 250–300 μm thickness, 6 days post-seeding [77]. Additionally, other studies have also investigated VSMCs seeding on naturally derived vascular scaffolds, such as decellularized matrices [78]. Knox et al. combined a decellularization approach of ovine arteries in a pulsatile flow bioreactor with cardiovascular progenitors to generate a biomimetic vascular graft [79]. In this setup, differentiated SMCs were observed by detection of calponin and MYH11 at the end of 3 weeks in culture [79]. Even though decellularized scaffolds possess many desirable characteristics for vascular replacements, such as niche-like architecture, mechanical properties and biochemical signals, they also entail some degree of structural weaknesses, such as low porosity and watertightness, hampering the possibility of direct VSMCs injection into the vessel wall [80]. In an attempt to develop bioengineered vessels with a more physiologically relevant SMC layer, a modular bioreactor and perfusion system were established to specifically allow for the proliferation of vascular-like constructs over extended culture periods [78]. Using a decellularized porcine artery as a model scaffold, the authors improved bioreactor design, including multiple contiguous functions contained within a single system, where tissue processing, cell seeding, and cell culture were developed to achieve clinically relevant constructs. However, in this study, VSMCs were unable to penetrate beyond the adventitial–medial boundary [78]. In this context, aiming to improve this intrinsic problem, Yazdani et al. designed a strategy consisting of cyclic bioreactor preconditioning (i.e., flow and pressure), combining it with surface modification (removal of the adventitial layer) of decellularized porcine carotid arteries [80]. This prompted an increase in cellular seeding efficiency and proliferation, thus promoting a more uniform deposition and density of mature VSMCs in the engineered vessel [80]. 

**Table 1:** Overview of relevant studies using different cell sources for SMC engineering.

Cell Source		Culture Setup (Scaffold; Cell Sorting; Growth Factors)	Main Outcome(s)	Reference
iPSCs		Alginate	High VSMC purity (>80%) and yield (~5.0 × 10^8^ cells/mL)	[60]
		Embryoid-based differentiation Normoxia vs. hypoxia (5%O_2_)	Higher VSMC viability under hypoxia	[61]
		Human platelet lysate and human serum vs. FBS culture medium supplementation	Mechanical strength comparable to hiPSC-derived VSMCs under FBS-supplemented medium	[58]
		Cell sorting for CD34+ cells PDGF-BB for SMCs differentiation	In vivo injection of SMCs into mouse models of urinary incontinence: tissue remodeling with higher detection of elastin	[65]
MSCs	ASCs	Low FBS supplementation	Identification of SMCs markers	[72]
		PLGA microcarriers	SMC-like phenotype, with cell attachment and proliferation on the microcarriers	[69,70]
		Collagen microspheres PDGF-AB and TGF-β1 Mechanical stretch	Identification of SMCs markers	[71]
		ASCs spheroids Bioprinting on gelatin–alginate TGF-β for SMCs induction	Assessment of viability, proliferation and SMC differentiation post-bioprinting	[81]
Primary SMCs		Polyurethane porous sheet-type scaffold Cyclic mechanical strain	Observation of SMCs contractile capacity	[73]
		Poly (lactide-co-caprolactone) electrospun scaffold and collagen coating Pulse bioreactor	Identification of α-SMA, and SMCs presented higher proliferation rates under pulsatile flow than when compared to static culture	[76]
		Electrospun gelatin fiber scaffold in bioreactor	Dynamic setup led to higher cell proliferation than under static conditions	[77]
		Decellularized porcine artery in bioreactor	High VSMC viability, variable levels of cell seeding in the decellularized matrices	[78,80]
		Nanofibrous gelatin–PLLA scaffold	In vivo model of urethral reconstruction exhibited SMC remodeling upon transplantation	[82]
		Bilayer silk scaffold	SMC alignment and proliferation in the scaffold	[83]
		Electrospun PCL and GelMA	High cell viability and proliferation	[84]
		Bioprinting on GelMA and hyaluronic acid	Bilayer of outer SMCs and inner ECs, with 20 mm length and 4 mm lumen diameter	[85]
Cardiovascular progenitor cells		Decellularized ovine arteries in a pulsatile flow bioreactor	VSMCs by detection of calponin and MYH11	[79]

ASCs: adipose-derived stem cells; ECs: endothelial cells; FBS: fetal bovine serum; GelMA: gelatin methacrylate; iPSCs: induced pluripotent stem cells; MSCs: mesenchymal stromal cells; MYH11: myosin heavy chain-11; PCL: poly(ε-caprolactone); PDGF: platelet-derived growth factor; PLGA: poly (D,L lactic-co-glycolic acid); PLLA: poly(l-lactic acid); SMCs: smooth muscle cells; TGF: transforming growth factor; VSMCs: vascular smooth muscle cells; α-SMA: alpha-smooth muscle actin.

When considering heterotypic culture configurations, it has been reported that in vitro vascular ECs–SMCs cocultures have an effect on SMC proliferation, migration, phenotypic expression and ECM production [86]. ECs can modulate SMC expression profile by increasing the expression of vascular endothelial cell growth factor (VEGF), PDGF-AA, PDGF-BB and TGF-β genes, and decreasing basic fibroblast growth factor (bFGF or FGF-2) gene expression, compared to SMC culture alone [87]. As such, these insights can be applied to improve tissue engineering strategies. Also, in the context of SMCs–ECs cocultures, in a study by Williams et al., a vascular construct perfusion bioreactor was developed, allowing for direct contact between SMCs and ECs, while providing a biomechanical environment that mimics features of the in vivo hemodynamics [88]. In this design, the bioreactor contained tubular poly (glycolic acid) (PGA) nonwoven felts, which were seeded sequentially with SMCs and ECs under dynamic flow conditions. SMCs populated the porous biomaterial and ECs were seeded onto the lumen surface, allowing the two cell types to interact. The lumen was perfused with culture medium, and ECs were exposed to pulsatile shear forces, mimicking the native aortic vessel wall tissue conditions. Results showed significant increase in SMC proliferation, more uniform cell distribution, more contractile SMC phenotype and downregulation of ECM deposition in the long term, compared to short-term coculture constructs [88]. 

Taken together, there is increasing interest in engineering SMCs in vascular grafts, using different types of natural-derived (silk protein) or synthetic scaffolds (polycaprolactone). In several studies, which differ in multiple parameters, such as cell origin, culture reagents, and scaffold design, mechanical stimuli seem to be a critical feature towards an SMC phenotype [71,89,90].

### 3.2. SkMCs

Different tissue sources have been explored to obtain SkMCs, not only by direct isolation of satellite and/or progenitor cells, but also by myogenic induction of MSCs (isolated from adipose tissue—ASCs, bone marrow and umbilical cord), and iPSCs [17]. To the best of our knowledge, in what concerns clinical translation, only cells obtained from SkM and adipose tissue have been applied in human clinical studies, which is covered in Section 4.

Concerning MSCs, several animal models (mouse, rat or larger models, like pig) have been employed as recipient models of muscle damage to study labelled donor MSCs [91,92]. In these studies, it was shown that bone marrow-derived MSCs can be recruited to areas of muscle injury and directly participate in myogenic regeneration by differentiation into myoblasts [91,92]. MSCs derived from other sources, namely umbilical cord, synovial membrane, adipose tissue, or tonsil, have been also shown to support muscle regeneration in vivo [93,94,95]. Particularly, concerning the umbilical cord, MSCs from Wharton’s jelly exhibited higher myogenic differentiation potential compared to cells isolated from umbilical cord blood under in vitro differentiation conditions, which seem to be related to CD90 basal expression by MSCs [96]. Under static culture conditions, MSCs can differentiate into SkM through stimulation with growth factors, typically FGF-2 and PDGF-AA [91], and others not so commonly reported, such as hepatocyte growth factor (HGF) and insulin-like growth facor-1 (IGF-1) [96]. On the other hand, in more complex in vitro 3D models, growth factor supplementation with HGF and IGF-1 was not sufficient to induce MSC myogenic differentiation [97]. MSCs are also extensively recognized by their paracrine secretion, potentially supporting regenerative features in a therapeutic context. For instance, in vivo models have described the proangiogenic potential of MSCs, providing a permissive niche for muscle regeneration [98,99]. Nonetheless, the potential of MSCs as a source of SkMCs for tissue engineering strategies is not well established. In the preliminary work by Testa et al., MSC differentiation towards SkMCs was tested using human serum counterparts instead of FBS [100]. Increased levels of myogenesis markers were observed when using high serum- or platelet-rich plasma concentrations, namely features restricted to the detection of CD56 and myosin proteins [100].

ASCs, in particular, have demonstrated the ability to differentiate into the myocyte lineage [101]. This was shown not only by identification of MyoD and other myogenic transcription factors, but also morphologically, by formation of multinucleated myofibers by myogenic induction of ASCs. ASC differentiation into myogenic lineage can be achieved through culture medium supplementation (e.g., FGF-2 and GSK3 inhibitor), and increased using biophysical cues, namely cyclic strain [101,102]. In animal models, ASCs seemed to accelerate muscle repair despite the fact that donor ASCs were not detected in recipient damaged muscle, pointing towards a paracrine action [103]. Moreover, a long-term effect on muscle regeneration was not observed [103]. On the other hand, in vivo local or systemic administration of myogenic progenitors derived from ASCs resulted in long-term engraftment (12 weeks post-transplantation) in a mouse model of DMD [101]. In another study, MyoD expression was induced in ASCs through lentiviral transduction, and engineered cells were administered into murine models of injured muscle, which effectively incorporated into the multinucleated fibers in the newly repaired muscle [104]. 

The use of hiPSCs to obtain SkMCs has been challenging [16,105] and the field was recently reviewed by Iberite et al. [106]. Protocols towards SkM differentiation have been reported based on gene transfer and external factors medium supplementation (growth factors and small molecules) [16]. hiPSC reprograming through gene transfer, for instance, based on viral vectors, aims to modulate overexpression of essential myogenic transcription factors previously mentioned, namely Pax [107] and MyoD [108]. This type of strategy may be able to produce cells at high yields, although the requirement of viral vectors can hinder cell therapy and tissue engineering prospects due to potential safety issues and differentiated cells might display an immature phenotype [16]. 

Differentiation of hiPSCs towards SkMCs has also been reported through modulation of culture conditions, especially through GSK3 β inhibitors and FGF-2 supplementation [109,110,111], and also the use of ascorbic acid, BMP-4, IGF-1, insulin and PDGF [112]. Indeed, modulation of multiple signaling pathways (Wnt, TGF- β, Notch, BMP, PI3K, hedgehog and retinoic acid) has been described, adding extra challenges towards robust and consistent platforms to obtain SkMCs [105,112,113]. The common feature between the multiple protocols reported is the production of Pax7^+^ myogenic progenitors, although at different efficiencies [110,112,114]. Moreover, in order to enrich for myogenic progenitors, FACS has been applied, based on different surface markers according to the protocols used, which in turn also differ and are influenced by culture parameters [16,115]. In fact, the surface markers reported varied between ERBB3^+^NGFR^+^ [109,115]; CD24^-^CD10^+^ [116]; CD57^−^ACHR^+^cMET^+^ [117]; and CD57^−^ NCAM1^+^ [118]. To facilitate the FACS protocol, a Pax7-based reporter system was tested [119]. It was observed that transplantation of the enriched population of Pax7 transgene expression myogenic progenitors resulted in cell engraftment into the murine muscle, while injection of a mixed population without previous FACS enrichment did not produce myofibers into the damaged muscle [119]. Although this type of knowledge acquired from murine models is valuable, its application in a human regenerative medicine context is limited, as more studies are needed to clarify and validate these markers in human counterparts. Overall, the purification protocols for target cell populations seem to enrich for muscle precursors that can exhibit enhanced myotube formation in vitro or muscle engraftment in vivo. Moreover, although iPSC differentiation protocols can allow for the generation of high numbers of myogenic progenitors (e.g., 2 × 10^16^ cells upon 43 days of culture [120]), there is still a need to understand the role and representativeness of the surface markers employed when using cell purification strategies and its correlation with cell function and therapeutic potential [16]. 

**Table 2:** Overview of relevant studies using different cell sources for SkMC engineering.

Cell Source		Culture Setup (Scaffold; Cell Sorting; Growth Factors)	Main Outcome(s)	Reference
iPSCs		GSK3 β inhibitor, FGF-2, ascorbic acid, BMP-4, IGF-1, insulin and PDGF [112]	Generation of Pax7+ myogenic progenitors at varying efficiencies	[112,114]
		GSK3 β inhibitor, FGF-2 and ITS	2 × 10^16^ myogenic progenitors upon 43 days of culture	[120]
		FACS based on different surface markers: ERBB3^+^NGFR^+^; CD24^−^CD10^+^; CD57^−^ACHR^+^cMET^+^; CD57^−^ NCAM1^+^	Enhanced in vitro myotube formation and/or enhanced muscle engraftment in vivo	[115,116,117,118]
		PCL scaffold with decellularized skeletal ECM motifs	In vivo cell integration in murine model of VML	[121]
MSCs	Adipose tissue	GSK3 β inhibitor and FGF-2 supplementation Cyclic strain	Identification of myogenic transcription factors and multinucleated myofibers	[101,102]
	BM	FGF-2 and PDGF-AA	Observed differentiation towards myoblasts	[91]
		HGF and IGF-1	HGF and IGF-1 may not be sufficient for myogenic differentiation	[97]
	Umbilical cord	Horse serum-supplemented medium or HGF, IGF-1 and FGF-2	Enhanced myogenic differentiation potential of MSCs from umbilical cord tissue compared to MSCs from umbilical cord blood	[96]
	Primary muscle	Human serum vs. FBS	Enhanced detection of CD56 and myosin proteins under high serum or platelet-rich plasma	[100]
Primary SkMCs		FACS depletion for CD45, CD31, CD11b, Sca1 and enrichment for CD34 and/or integrinα7 Fibrin vs. Matrigel	Higher cell expansion (threefold) on fibrin gel than Matrigel	[122]
		Electrospun nanofiber of PMMA	Laminin-coated PMMA facilitated myoblast proliferation in comparison to collagen-coated PMMA	[123]
		Collagen and Matrigel replating steps	Short-time protocol with cell isolation from low amounts of skeletal muscle (0.02 g)	[124]
		Fibrin-based hydrogel	Electrical stimulation led to improved contractility and mature phenotype	[125]

BMP: bone morphogenetic protein; ECM: extracellular matrix; FACS: fluorescence-activated cell sorting; FBS: fetal bovine serum; HGF: hepatocyte growth factor; IGF-1: insulin-like growth facor-1; iPSCs: induced pluripotent stem cells; ITS: insulin–transferrin–selenium–ethanolamine; MSCs: mesenchymal stromal cells; PCL: poly(ε-caprolactone); PDGF: platelet-derived growth factor; PMMA: poly(methyl methacrylate); SkMCs: skeletal muscle cells; VML: volumetric muscle loss.

Coculture strategies have been reported to recapitulate more closely the native SkM environment. Cocultures of isolated myoblasts with fibroblasts seem to improve the migration of the myogenic cells, but no effects on proliferation or myotube formation were explored [44]. Other authors reported cocultures of primary SKMCs with MSCs to improve myogenic differentiation and did not observe a significant effect on muscle cell proliferation [126,127]. Particularly, in a study by Cai et al., cocultures of primary rat myoblasts with MSCs from bone marrow or adipose tissue were performed on electrospun polycaprolactone and collagen scaffolds [127]. Under differentiation conditions for myoblasts (a low serum concentration (2%) and a serum-free formulation), it was possible to observe an upregulation of myogenic markers such as myosin heavy chain 2 and α-actinin 2 on the coculture settings in comparison to monocultures [127]. On the other hand, in a study by Juhas et al., an immune cell population, macrophages, was included into engineered constructs of murine myoblasts within Matrigel and fibrin [128]. Promotion of myogenesis and less myofiber apoptosis were observed after induced chemical muscle injury [128]. Interestingly, other authors reported the use of inflammation-related cytokines to improve in vitro satellite cell culture and in vivo muscle engraftment in a recipient mouse model [129]. Moreover, the use of conditioned medium from other immune cells, T lymphocytes, seemed to sustain satellite cell proliferation to higher cell passages [130].

Attempts to expand satellite cells ex vivo have been described in multiple studies, whether by inhibiting the production of differentiation factors or by promoting their proliferative capacity [55,131]. Of note, culture parameter specifications required to promote human myoblast proliferation and prevent spontaneous differentiation during the expansion phase may include close monitoring of cell confluence levels and specific coatings, such as those laminin-based [132,133]. 

Besides the modulation of biochemical conditions through different culture medium cocktails, physical cues are also critical to SkMCs. For example, soft hydrogels with elastic modulus similar to muscle tissue were shown to sustain self-renewal of satellite cells better than plastic surfaces [134]. Importantly, two frequently used biomaterials, collagen, usually derived from rat tail, and Matrigel, represent xenogeneic options to be used in this context [17]. Zhu et al. also used xenogeneic fibrin in comparison to Matrigel to culture murine SkMCs after tissue digestion with collagenase and dispase, and FACS enrichment [122]. Cells negative for CD45, CD31, CD11b, Sca1 markers, and positive for CD34 and/or integrin-α7 were considered muscle stem cells and a threefold higher cell expansion factor was reported when using fibrin gel compared to Matrigel. The use of biomaterials in SkM engineering has been extensively reviewed [135] and there is a huge search for matrices that can mimic the native ECM and provide structural architecture with the mechanical signals offered by the scaffold supporting satellite cell maintenance [136]. 

Overall, there is still an unmet need towards the development of culture systems amenable to recapitulate the native tissue microenvironment ex vivo in order to support the self-renewal of satellite cells and the maintenance of their regenerative ability through the generation of differentiated cells with a contractile function [17].

## 4. Clinical Studies with Expanded SMCs and SkMCs

Despite the multiple efforts performed towards muscle regenerative approaches, there is still a limited number of human clinical trials studying the administration of SMCs or SkMCs. Importantly, upon transplantation into damaged tissues, the administered cells (SMCs or SkMCs) face unfavorable environment signals (e.g., limiting oxygen concentrations), which hinders their maintenance, proliferation and function [137]. Concerning SkM, stem/progenitor cells have shown the potential to restore injured muscle, although it has been observed that their capacity ex vivo is lost, as aforementioned. 

Table 3 summarizes the main clinical trials employing distinct types of muscle cells in the autologous context and for different pathological settings, with functional incontinence being one of the major applications. The required cell number of SMCs or SkMCs varied among the studies analyzed, but included multiple doses with a range from 5 to 200 million cells per administration [138]. Of notice, several clinical trials employed ASCs as a therapeutic approach for stress urinary incontinence [139,140,141]. Despite the fact that the use of ASCs could potentially overcome the limited efficiency of the ex vivo expansion of SkMCs and SMCs, a trial with five female patients did not show any effective improvement [139]. Overall, ASCs hold great promise as a cell source for muscle repair and regeneration, but further studies are needed to establish their effectiveness. In general, most of the trials showed safety (phase I) and preliminary efficacy (phase I/II) but are limited in terms of objective measurements of successful outcome.

## 5. Conclusions 

The wide distribution of SM and SkM throughout the body, along with their unique properties, highlights the importance of in-depth knowledge of their anatomy, physiology and function. This understanding is crucial to addressing clinical needs in cases of organ or tissue damage.

A critical aspect in the context of muscle tissue engineering is the limited availability and suitability of donor tissues for cell harvesting. Autologous cells, for instance, might display impaired regenerative potential due to a specific pathological setting and/or aging. To overcome this challenge, alternative cell sources, such as iPSCs or adult stem/progenitor cells like MSCs, have been proposed as a starting source for myogenic differentiation, particularly for SMC bioengineering [58,59,69]. Similar considerations apply to SkMC engineering, in which tissue resident stem cells (or satellite cells) represent a potential cell source, though with challenges, especially when considering elderly patients [17,51]. 

Bioreactors are crucial tools for the robust development of standardized and high-quality engineered tissue products, with great potential to advance therapies targeting muscle repair and regeneration. Indeed, bioreactor technologies can be used to (i) expand the target cell types to generate clinically relevant cell numbers and/or (ii) mimic the in vivo microenvironment by exposing cells to relevant physical and biochemical stimuli allowing for the generation of cells and tissues with the desired identity and function. Of note, the development of engineered muscle tissues also relies on the use of biomaterials able to support tissue function, and more complex approaches, such as bioprinting, are being explored [153,154].

**Figure 2:**
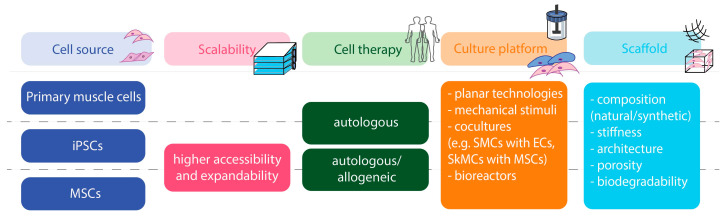
Considerations on the key points for SMC and SkMC manufacturing. From selection of cell source to culture setup, with potential combination of biomaterials. In terms of cell source, MSCs and iPSCs present broader availability and higher expansion potential, though with a limited maturation phenotype of the target cell type. Due to their low immunogenic profile, MSCs can be used in an allogeneic therapeutic setting, in contrast to primary isolated muscle cells. Concerning culture systems, a balance between complexity (e.g., cocultures that need to accommodate more than one cell type) and feasibility must be considered, with mechanical stimulus representing a critical aspect in muscle tissue engineering. Scaffold design and selection should take into account multiple characteristics such as its composition and stiffness, and if it targets in vitro/ex vivo use only, or if it is intended to be used as a cell delivery vehicle in vivo or as an architectural implanted scaffold. Adapted from [155,156,157]. ECs: endothelial cells; iPSCs: induced pluripotent stem cells; MSCs: mesenchymal stromal cells; SkMCs: skeletal muscle cells; SMCs: smooth muscle cells.

Overall, the translation of engineered muscle tissue-based strategies into clinics depends on biotechnology advances involving the combination of innovative cell culture technologies and biomaterial scaffold fabrication, followed by the optimization of large-scale manufacturing processes (Figure 2). Importantly, tissue engineering-based approaches are classified as advanced therapy medicinal cell products (ATMPs) by the European Medicines Agency (EMA). Thus, both biomaterial and cellular components must undergo rigorous quality control and detailed certification to meet good manufacturing practice (GMP) criteria. This entails the need for directing strategies from the very beginning of the experimental setup towards translation in order to establish muscle tissue engineering grafts as the future gold standard for muscle repair and regeneration.

## Figures and Tables

**Table 3:** Summary of cell types used in major clinical trials targeting muscle repair and regeneration.

Cell Type	Culture Conditions	Disease/Application	Number of Patients	Main Observations	Year	Reference
Myoblasts	-	Duchenne muscular dystrophy	8	No dystrophin expression restored; No significant strength recovery.	1992	[142]
Myoblasts	DMEM with 10% horse serum	Duchenne muscular dystrophy	21	No significant strength recovery.	1992	[143]
Myoblasts	FBS and FGF-2	Oculopharyngeal muscular dystrophy	12	Safety tested; Quality of life improved.	2014	[144]
SkM-derived cells	Proprietary method	Urinary incontinence	38	Safety tested; No major adverse events.	2013	[145]
SkM-derived cells	Proprietary method	Urinary incontinence	80	Safety profile tested; Improvement in stress incontinence symptoms.	2014	[138]
SkM-derived cells	Ham’s F10 with 20% FBS	Urinary incontinence	20	Partial response; Symptoms relapse after 2 years in 50% of the initial responders.	2019	[146]
SkM (minced tissue)	Without ex vivo culture	Urinary incontinence	35	Symptoms improved in 25–63% of patients; Minor adverse events.	2014	[147]
SkM-derived cells	DMEM/F12 and 10% fetal calf serum	Damaged urethral sphincter	222	After 1 year, in 46% patients, no therapeutic effect; 42% reported improvement of symptoms and in 12% urinary continence was restored.	2012	[148]
Myoblasts (and fibroblasts)	DMEM/F12 with 20% autologous serum	Urinary incontinence after prostatectomy	63	1 year follow-up shows restored continence in 41 patients; 5 patients did not show improvement.	2008	[149]
Progenitor SkM cells	-	Urinary incontinence	12	Quality of life improvement reported.	2010	[150]
SMCs (and urothelial cells)	DMEM with 10% fetal calf serum	Cystoplasty	7	56% of patients showed signs of enhanced bladder function.	2006	[151]
SMCs (and epithelial cells)	DMEM with EGF	Urethral reconstruction	5	Histology showed that engineered constructs integrated patient’s tissue.	2011	[152]

DMEM: Dulbecco’s modified Eagle’s medium; FBS: fetal bovine serum; EGF: epidermal growth factor; FGF: fibroblast growth factor; SMCs: smooth muscle cells; SkM: skeletal muscle.
